# Polysaccharide Responsiveness Is Not Biased by Prior Pneumococcal-Conjugate Vaccination

**DOI:** 10.1371/journal.pone.0075944

**Published:** 2013-10-11

**Authors:** Jens Magnus Bernth-Jensen, Ole Schmeltz Søgaard

**Affiliations:** 1 Department of Clinical Immunology, Aalborg Hospital, Aarhus University Hospital, Aarhus, Denmark; 2 Department of Infectious Diseases, Aarhus University Hospital, Aarhus, Denmark; University of Ottawa, Canada

## Abstract

Polysaccharide responsiveness is tested by measuring antibody responses to polysaccharide vaccines to diagnose for humoral immunodeficiency. A common assumption is that this responsiveness is biased by any previous exposure to the polysaccharides in the form of protein-coupled polysaccharide vaccines, such as those used in many childhood vaccination programmes.

To examine this assumption, we investigated the effect of protein-coupled polysaccharide vaccination on subsequent polysaccharide responsiveness.

HIV-infected adults (n = 47) were vaccinated twice with protein-coupled polysaccharides and six months later with pure polysaccharides. We measured immunoglobulin G responses against three polysaccharides present in only the polysaccharide vaccine (non-memory polysaccharides) and seven recurring polysaccharides (memory polysaccharides). Responsiveness was evaluated according to the consensus guidelines published by the American immunology societies.

Impaired responsiveness to non-memory polysaccharides was more frequent than to memory polysaccharides (51% versus 28%, P = 0.015), but the individual polysaccharides did not differ in triggering sufficient responses (74% versus 77%, P = 0.53). Closer analysis revealed important shortcomings of the current evaluation guidelines. The interpreted responseś number and their specificities influenced the likelihood of impaired responsiveness in a complex manor. This influence was propelled by the dichotomous approaches inherent to the American guidelines. We therefore define a novel more robust polysaccharide responsiveness measure, the Z-score, which condenses multiple, uniformly weighted responses into one continuous variable. Using the Z-score, responsiveness to non-memory polysaccharides and memory-polysaccharides were found to correlate (R^2^ = 0.59, P<0.0001).

We found that polysaccharide responsiveness was not biased by prior protein-coupled polysaccharide vaccination in HIV-infected adults. Studies in additional populations are warranted.

## Introduction

Polysaccharide responsiveness is tested by measuring antibody responses to polysaccharide vaccines. The test forms a cornerstone in diagnosing humoral immunodeficiency [1]–[4]. However, the basis for the test appears to be eroding. The reason is that childhood vaccination schemes now commonly include the same polysaccharides, which are used for testing, but in protein-coupled form. This may be problematic because the dominant perception in the field is that all future responses to protein-coupled polysaccharides - even without the protein moiety – will describe protein responsiveness rather than polysaccharide responsiveness [5]. Thus, it is difficult to test polysaccharide responsiveness *and* to acknowledge the dominant perception. The key question is to which extent the dominant perception is correct.

Indeed, protein-coupling of polysaccharides alters the immune response, e.g. IgG1 and IgG3 predominate over IgG2, and efficient long-lasting immunologic memory is induced [6]. The simplistic explanation model is that the protein-moiety enables T-cell synergy. However, the impact of previous exposure to protein-coupled polysaccharide vaccination on the outcome of polysaccharide responsiveness test remains unaddressed. Clarification of this open question is of basal immunological interest and of immense importance to clinical practice in the field.

We aimed to study protein-coupled polysaccharide vaccinatiońs potential bias upon testing polysaccharide responsiveness in HIV-infected adults. No bias was identified.

## Methods

### Ethics statement

This observational study was a sub-study to an investigator initiated phase Ib/IIa, randomized, double-blind, placebo-controlled trial, in which HIV-infected adults were immunised with pneumococcal vaccines with or without CPG 7909. We have previously detailed the populatiońs data [7].

HIV patients seen at the outpatient clinic of the Department of Infectious Diseases, Aarhus University Hospital, Denmark were invited by letter to participate. Consenting HIV-seropositive volunteers aged 18 or older were eligible for enrollment. All participants gave informed written consent. The study protocols were approved by the Danish Medicines Agency, the Regional Ethical Committee, and the Danish Data Protection Agency; and the protocols were registered at www.clinicaltrials.gov (NCT00562939).

### Study population

**Figure 1:**
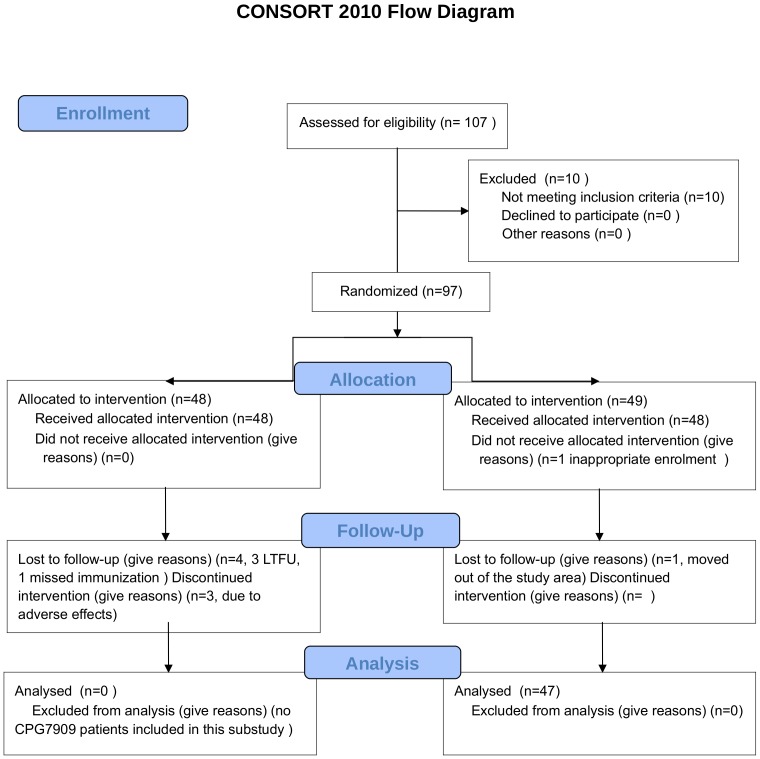
Flow diagram illustrating patient inclusion.

Briefly, we excluded individuals who (i) had received pure pneumococcal polysaccharide vaccine within the last 5 years; (ii) were on antiretroviral therapy for less than 6 months or with HIV RNA >50 copies/mL; (iii) with CD4+ cell count <200 cells/µL; (iv) were considered unable to follow the protocol regimen; (v) were women who were pregnant, breast-feeding, or unwilling to use reliable contraception methods for the duration of the trial. In the present study, we included the 47 participants who received no CPG 7909 (**Figure 1**). Participants were immunized with double doses of protein-coupled pneumococcal polysaccharides (Prevenar7) at 0 and 3 months, and pure pneumococcal polysaccharide vaccine (Pneumovax) at 9 months during the study period.

Plasma samples used in this study were obtained at 3, 9, and 10 months. IgGs to ten polysaccharides contained in the pure polysaccharide vaccine were quantified by ELISA at Statens Serum Institute, Copenhagen, Denmark according to WHO recommendations. Seven of the ten polysaccharides were also contained in the protein-coupled vaccine (PS4, PS6B, PS9V, PS14, PS18C, PS19F, and PS23F). These seven polysaccharides are referred to as memory-polysaccharides. Three of the ten polysaccharides were only contained in the polysaccharide vaccine (PS1, PS7F, and PS19A). These three are referred to as non-memory polysaccharides.

### Interpretation of polysaccharide responsiveness

Initial interpretation was made based on expert consensus guidelines published by the American Academy of Allergy, Asthma, and Immunology, the American College of Allergy, Asthma and Immunology, and the Joint Council of Allergy, Asthma and Immunology [1]. Sufficient response to an individual polysaccharide was defined as a post-vaccination specific IgG concentration ≥1.3 µg/mL *or* a ≥ four-fold increment over baseline. Impaired polysaccharide responsiveness was defined as less than 70% of individual polysaccharide responses being sufficient.

Four alternative approaches for interpreting polysaccharide responsiveness were explored. These approaches were all based on post-vaccination IgG concentration to polysaccharides (concentrations). 1) Geometric mean concentrations were calculated and impaired responsiveness was defined as values below 1.3 µg/ml. 2) Geometric mean concentrations where individual concentrations were corrected for population differences between polysaccharides. This correction was done by dividing individual concentrations with the populatiońs geometric mean concentration to the particular polysaccharide. Impaired responsiveness was defined as values below 0.35 (1.3µg/ml divided by the populatiońs geometric mean of all concentrations to all polysaccharides (3.7µg/ml)). 3) Variance differences between responses were corrected by transforming individual responses to standard normal deviates. These were then combined by arithmetic means (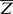). Each standard normal deviate (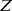) was calculated from a logarithmically transformed concentration (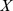), corrected by the populations mean (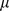) and SD (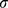) of logarithmically transformed concentrations to the particular polysaccharide:
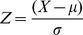



Impaired responsiveness was defined as 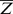 below –0.79 (the logarithm of 0.35 divided by the SD of all logarithmically transformed responses (0.57)). 4) Number of 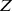 ´s importance for 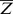
's variance was corrected by transforming 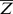 to a standard normal deviate (Z-score). This was done by dividing each 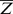 by the populatiońs corresponding SD. Impaired responsiveness was defined as a Z-score below –0.79.

### Simulation

All data on responses were included. Responses were organised in unique panels. Each panel contained a variable number of polysaccharide responses; from 1 to 9 out of the total 10. For a given number of responses (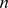), we established all possible unique panels by including the possible combinations of the available 10 polysaccharides responses (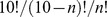). The populatiońs frequency of impaired responsiveness was determined with each unique panel. Panels were grouped by the number of included polysaccharide responses and the mean frequency and SD of impaired responsiveness were calculated each group. The simulations were made in Excel spreadsheets (Microsoft® Office Excel® 2007).

Paired data were tested for difference by Wilcoxon matched-pairs signed-ranks test. Outcome concordance was tested by Fisher's exact test. IgG concentrations were log-normally distributed. To test polysaccharide responses for differences (individually and simultaneously), observations were coupled to dummy-variables indicating their origin. Linear regression and F tests were used for logarithmic transformed IgG concentrations whereas probit regression and chi-squared testing were used for dichotomous variables. Correlation of Z-scores was examined by linear regression. Significance level: P<0.05. Data analysis was made using Stata 11.0 (StataCorp, TX).

## Results

### Responsiveness to memory and non-memory polysaccharides is concordant

We studied HIV-infected adultś responsiveness to two polysaccharide groups: polysaccharides to which immunological memory was anticipated (*memory polysaccharides*) and polysaccharides to which immunological memory was not anticipated (*non-memory polysaccharides*). Responsiveness was evaluated based on American consensus guidelines [1]: An adequate response to an individual polysaccharide was defined as a post-immunisation IgG concentration of at least 1.3 µg/ml or at least four fold over baseline and a person was defined as having impaired responsiveness if less than 70% of individual responses were adequate. A total of 28% had impaired responsiveness to memory polysaccharides, whereas 51% had impaired responsiveness to non-memory polysaccharides (P = 0.015). However, the test outcome (*impaired responsiveness* or *not impaired responsiveness*) was concordant in 64% of the participants (P = 0.049). We therefore rejected independency of responsiveness to memory and non-memory polysaccharides.

According to consensus guidelines, a pre-vaccination IgG concentration *above* 1.3 µg/ml entails an insufficient response if the post-vaccination IgG concentration is *below* 1.3 µg/ml. We examined the consequences of also accepting pre-vaccination concentrations above 1.3 µg/ml as sufficient responses. Now, 23% had impaired responsiveness to memory polysaccharides, whereas 49% had impaired responsiveness to non-memory polysaccharides (P = 0.005). The test outcome was concordant in 66% of the participants (P = 0.017). Thus, the additional definition did not substantially alter the findings.

### Memory and non-memory polysaccharides do not differ in triggering sufficient responses

We speculated that the unequal frequency of impaired responsiveness to memory polysaccharides and non-memory polysaccharides could be explained by an unequal propensity to trigger sufficient responses. However, no difference could be demonstrated; 77% of the responses to memory polysaccharides were sufficient and 74% of the responses to non-memory polysaccharides were sufficient (P = 0.53). Essentially similar findings were obtained, when we accepted pre-vaccination concentrations above 1.3 µg/mL as sufficient responses (79% versus 75%, P = 0.45).

### Number and specificity of responses decide impaired responsiveness frequency

**Figure 2:**
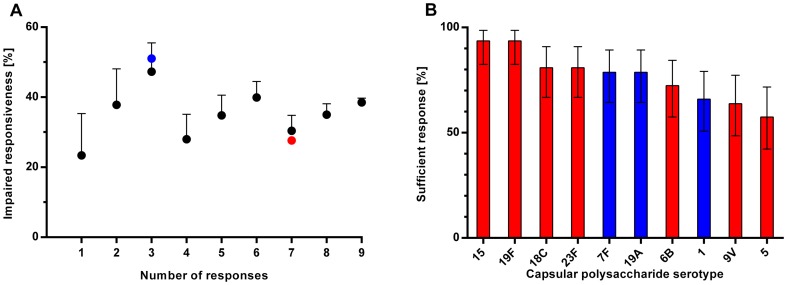
Polysaccharide responsiveness depending on the number of tested responses and their specificities. **A:** Participantś polysaccharide responsiveness was evaluated according to consensus guidelines from a variable number of responses (one to nine). All combinations of response specificities were evaluated yielding a total of 1022 responsiveness evaluations per participant. Responsiveness was evaluated according to the consensus guidelines. The populations mean frequencies of impaired responsiveness are presented with SDs ordered according to the number of included responses. The particular frequencies for the three non-memory polysaccharides and seven memory polysaccharides are indicated (blue symbol and red symbol, respectively). **B:** The percentage attaining a sufficient response to the individual polysaccharides according to consensus guidelines. Blue bars represent responses to non-memory polysaccharides and red bars represent responses to memory polysaccharides.

We were puzzled by finding an unequally impaired responsiveness frequency to the two polysaccharide types *without* any demonstrable differences in their propensity to trigger sufficient responses. We hypothesised that the unequal numbers of interpreted responses (three versus seven) could explain this controversy. This hypothesis was tested in a simulation based on all available data. From **Figure 2A** is it clear that the number of responses interpreted immensely impacts a populatiońs impaired responsiveness frequency. Individual polysaccharides differed in their propensity to induce sufficient responses (**Figure 2B, **P = 0.0039) wherefore the choice of polysaccharide responses used for testing heavily influences the tests outcome.

The frequencies of impaired responsiveness based on responses to the seven memory polysaccharides and the three non-memory polysaccharides did not appear unusual because both were within 0.6 SD of their respective means (Figure 2A). However, impaired responsiveness is generally more frequent when any three responses are interpreted than when any seven responses are interpreted (**Figure 2A**, the causal relationship is given in **File S1** and **Figure S1**).

Thus, the more frequently impaired responsiveness from the three non-memory polysaccharides compared with the seven memory polysaccharides is most likely caused by their unequal number.

### Condensing polysaccharide responsiveness into one variable termed Z-score

It seems inappropriate that the specificity and number of responses decide the frequency of impaired responsiveness in a population. We therefore decided to develop a more reliable polysaccharide responsiveness measure to achieve our aim. We anticipated that a single continuous variable would be most suitable. This made it necessary to choose between post-vaccination concentrations *or* fold-increments. Post-vaccination IgG concentrations were above 1.3 µg/mL in 98% of the sufficient responses, and no test outcome was changed by abandoning this criterion. Thus, the "fold-increment" criterion was redundant and post-vaccination IgG concentrations were therefore used to describe responses in the remaining study.

**Figure 3:**
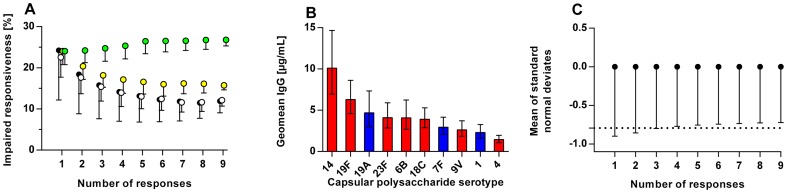
Alternative measures of polysaccharide responsiveness. **A:** Participantś polysaccharide responsiveness was evaluated using means of responses. A variable number of responses were included (one to nine). All combinations of response specificities were evaluated. The populations mean frequencies of impaired responsiveness for a given number of responses were determined from geometric means (black), concentration-corrected geometric means (white), means of standard normal deviates (yellow), means of standard normal deviates transformed to standard normal deviates (green). See **Methods** for impaired responsiveness definition. Error bars indicate SDs. **B**: Populations geometric mean IgG concentrations to polysaccharides after polysaccharide vaccination (with 95% confidence intervals). IgG concentrations to non-memory polysaccharides are presented as blue bars and IgG concentrations to memory polysaccharides are presented as red bars. **C**: Means of polysaccharide responsiveness based on standard normal deviates obtained from the simulation. Error bars indicate SDs. The arbitrary limit defining impaired responsiveness (–0.79) are illustrated for comparison.

To launch the development, the geometric mean of responses was used as responsiveness measure. Black symbols in **Figure 3A** display the relationship between the impaired responsiveness frequency and the number of interpreted responses. The populatiońs responses differed between polysaccharides (P<0.0001, **Figure 3B**). Correction of these differences caused decreases in SDs (**Figure 3A**, white symbols). The decreased SDs indicates lesser importance of which particular polysaccharide responses that are used to evaluate responsiveness. Next, the difference in polysaccharide response variation was also corrected by transforming responses to standard normal deviates before averaging (**Figure 3A**, yellow symbols). This procedure diminishes the dependency on the number of included responses but it did not abolish it. From **Figure 3C** is it evident that the mean SDs of the latter averages are inversely related to the number of included responses. This dependency was abolished by transforming the averages to standard normal deviates. This variable was termed Z-score. The expected frequency in a population having an impaired responsiveness is not dependent on the specificity and the number of responses included in the Z-score (**Figure 3A**, green symbols).

### Responsiveness to memory and non-memory polysaccharides correlates

**Figure 4:**
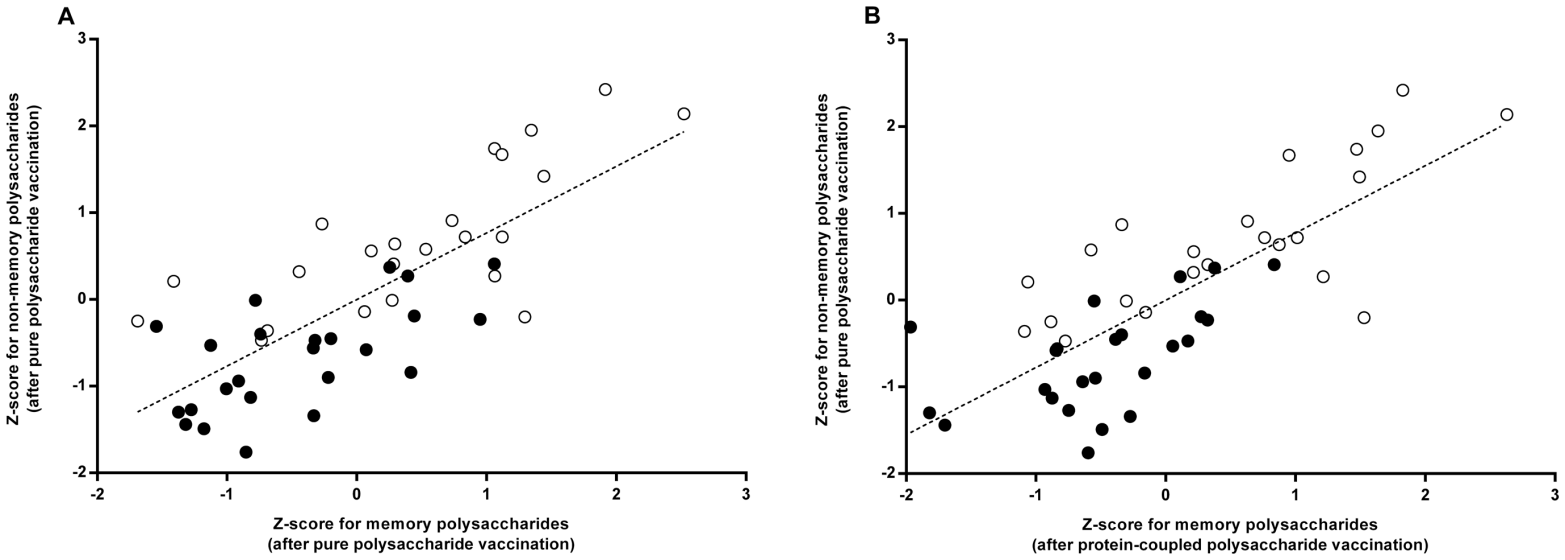
Correlation of Z-scores to memory polysaccharides and non-memory polysaccharides. Z-scores of responses to non-memory polysaccharides after polysaccharide vaccination were compared to Z-scores of responses to memory polysaccharides (**A**): after polysaccharide vaccination and (**B**): after protein-coupled polysaccharide vaccination. The Z-scores of participants with impaired polysaccharide responsiveness to non-memory polysaccharides according to consensus guidelines are presented as black symbols whereas the Z-scores of participants with intact responsiveness are presented as white symbols.

With Z-score in the armoury, we returned to complete the study of the potential bias of the protein-coupled polysaccharide vaccination to testing polysaccharide responsiveness. From **Figure 4A** is it clear that the polysaccharide vaccination triggered responsiveness to memory and non-memory polysaccharides correlated intimately (P(slope = 0)<0.0001, *R* ^2^ = 0.59). The correlation was observed both in persons with impaired non-memory polysaccharide responsiveness according to consensus guidelines (black symbols in **Figure 4A**, n = 24, P(slope = 0) = 0.0012, *R* ^2^ = 0.39) as well as in persons without impaired non-memory polysaccharide responsiveness (white symbols in **Figure 4A**, n = 23, P(slope = 0)<0.0001, *R* ^2^ = 0.55). The regression coefficients of these two participant subpopulations could not be demonstrated to differ (P = 0.59).

We further stressed the assumed bias introduced by protein-coupling. Responsiveness to protein-coupled polysaccharide vaccination (prior to pure polysaccharide vaccination) was compared to the responsiveness to pure polysaccharide vaccination. These responsiveness measures also correlate intimately (P(slope = 0)<0.0001, *R* ^2^ = 0.60, **Figure 4B**). Again, the correlation was observed both in persons with and without impaired non-memory polysaccharide responsiveness according to consensus guidelines (n = 24, P(slope = 0) = 0.0047, *R* ^2^ = 0.31 and n = 23, P(slope = 0)<0.0001, *R* ^2^ = 0.57). The regression coefficients of these two participant subpopulations could not be demonstrated to differ (P = 0.28).

## Discussion

Polysaccharide responsiveness has been presumed to be biased by prior exposure to pneumococcal-conjugate vaccines. In the present study, we investigated this presumption. We here present the first evidence that polysaccharide responsiveness is not biased by prior pneumococcal-conjugate vaccination.

We found that impaired responsiveness was more frequent to non-memory polysaccharides than to memory polysaccharides. This was contrasted by the surprising finding that memory and non-memory polysaccharides did not differ in terms of their propensity to trigger sufficient responses. We dissected this controversy and found it to be propelled by the dichotomous evaluation of multiple responses inherent in consensus guidelines. We deducted an alternative continuous polysaccharide responsiveness measure termed the Z-score. By using this score, we found that the responsiveness to non-memory polysaccharides correlated intimately with the responsiveness to memory-polysaccharides both when the latter were triggered by polysaccharides *and* by protein-coupled polysaccharides.

The main strength of the present study is the study population. HIV-infected persons are susceptible to pneumococcal disease [8] and frequently have impaired polysaccharide responsiveness [9]. The American consensus guidelines for evaluating polysaccharide responsiveness have been validated in HIV-infected persons [9]. Furthermore, we maximized the potential for bias by previous protein-coupled polysaccharide immunisation by administering double doses of protein-coupled polysaccharide vaccine 9 months and 6 months before testing polysaccharide responsiveness.

However, our study also had some limitations. Inclusion of a group not receiving protein-coupled polysaccharide vaccine could have contributed additional information to the present study. Vaccination with protein-coupled polysaccharides has been reported to prime responses to recurring polysaccharides in pure polysaccharide vaccines [10]–[12]. But this effect was not observed in other studies [13]–[15].

More responses to non-memory polysaccharides should ideally have been quantified; optimally, as many as to the memory polysaccharides. This would eliminate the impact of unequal response numbers (**Figure 2A**). But no more IgG specificities could be quantified by the used laboratory at the time of the study. Now, more responses are easily tested using fluorescent bead-based immunoassays [16]. However, the valency of the conjugate vaccine has also increased and now shares 13 polysaccharides with the pure polysaccharide vaccine. This development is likely to continue. Our findings in a population with high frequency of impaired polysaccharide responsiveness are therefore important.

Obviously, other populations must be studied before final conclusions are made. Different age groups are of special interest because the importance of protein-coupling differs [17]. With increasing age after infancy, responses to protein-coupled polysaccharide vaccines gradually display the characteristics expected of pure polysaccharide antigens; that is a relative increase in IgM and IgG2 compared to IgG1 [17].

Our findings suggest that the consensus guidelines used for evaluating polysaccharide responsiveness should be revised. The Z-score offers several advantages over the classic dichotomous interpretations of multiple individual responses. One variable is simpler to interpret. The Z-score is robust to difference in numbers and specificities of responses (**Figure 3A**). Lack of such robustness is, indeed, an Achilles heel of the American consensus guidelines (**Figure 2A**) which also makes comparison of studies treacherous except when exactly the same response specificities are tested. Furthermore, we expect that a continuous variable better describes the clinical continuum of polysaccharide responsiveness. Clinical use of the Z-score necessitates estimation of unknown figures (means and SDs) from a suitable reference population. Patientś polysaccharide responsiveness can then be presented as percentiles of the reference population. This alleviates the common limit-value used to define sufficient responses for all polysaccharides (1.3 µg/ml), which is not likely to be optimal.

The sacrifice in our alternative approach is that we abandoned the “fold-increment” criterion. In the present study, this criterion was redundant. In our opinion, the criterion is somewhat misleading. A tiny absolute response could satisfy the criterion if the baseline is also low, even though the response is abnormal and non-protective. Also, omitting the criterion entails less patient discomfort and savings.

How many response specificities should be included in the Z-score? Measuring responses to different polysaccharides is essentially analogous to making repeated measures of one variable because the individual responses correlate closely. Consistent with this, the SDs of standard normal deviate means were inversely related to the number of responses (**Figure 3C**). The decrease in SDs was negligible when more than three to four responses were included. Therefore, we expect inclusion of more response specificities in the Z-score to be superfluous.

Other approaches for evaluating polysaccharide responsiveness may also be forthcoming. A screening candidate could be αGal-binding IgG. αGal (Galα1,3Galβ1,4GlcNAc-R) is a microbial carbohydrate structure described as the most frequent ligand of human IgGs [18]. We recently reported that αGal-binding antibodies correlate with the classic measures of carbohydrate responsiveness; namely antibodies to blood type antigens A and B [19]. However, antibody concentration is one thing. Function may be another. Indeed, opsonophagocytic assays is stressed as the future of antibody testing [20].

Revised definitions of polysaccharide responsiveness are to be further explored. But, importantly, the advent of pneumococcal conjugate vaccines does not necessarily end the era of diagnostic pneumococcal polysaccharide vaccination.

## Supporting Information

Figure S1
**The probability of insufficient individual responses' impact on impaired responsiveness frequency.** Illustration of impaired responsiveness frequency in fictive populations with different *k*-values as functions of the numbers of responses examined. The presented *k*-values are products of the average *k*-value in the present study (0.23) multiplied by the following multiplicands: **A** = 1/2, **B** = 2/3, **C** = 1, **D** = 3/2, and **E** = 2). Impaired responsiveness frequency was calculated as the percentage of the population expected to respond insufficiently to at least 30% of polysaccharides (under the defined conditions). Thus, the percentage expected to have impaired responsiveness when number of responses belonging to “first step” (i.e. 1, 2, or 3 responses) is calculated as 1 minus the probability of producing maximal number of insufficient responses without qualifying for impaired responsiveness. Thus, on the “first step”: 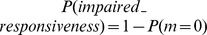 where *m* is the number of maximal allowed insufficient responses without qualifying for impaired responsiveness (on the first step no insufficient responses are allowed). An additional subtrahend is added for every subsequent “step”; namely 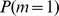 on the “second step” and 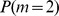 on the “third step” and so forth. Each subtrahend is given by: 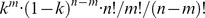 where *n* is the number of responses.(TIF)

File S1 (DOCX)
