# Supplementary material for: Polysaccharide Responsiveness Is Not Biased by Prior Pneumococcal-Conjugate Vaccination
Source: PLoS One. 2013 Oct 11;8(10):e75944. doi: 10.1371/journal.pone.0075944 (PMC3795730; doi:10.1371/journal.pone.0075944)
Supplement: File S1 — (DOCX) [file pone.0075944.s002.docx]

**Supporting Information file S1**

According to consensus guidelines, persons of age more than 5 years have impaired polysaccharide responsiveness if more than 30% of responses to individual polysaccharides are insufficient [1]. The *exact* number of responses to be examined is not defined which we claim undermines the usability of the consensus guidelines. The explanation is given here.

Common to examination of 1, 2, or 3 individual polysaccharide responses: one (or more) insufficient response will satisfy the criterion for impaired responsiveness because more than 30% of individual responses are insufficient. However, the risk of producing at least 1 insufficient response increases with the number of examined individual responses. Therefore, classification as “impaired responsiveness” is most likely when 3 responses are examined, less likely when 2 responses are examined and least likely when 1 response is examined.

Common to examination of 4, 5 or 6 responses: two (or more) insufficient responses will satisfy the criterion for impaired responsiveness. The risk of producing at least 2 insufficient responses increases with the number of examined responses. Therefore, “impaired responsiveness” is most likely when 6 responses are examined, less likely when 5 responses are examined and least likely when 4 responses are examined.

This relation continues in steps containing 3 consecutive numbers of responses. On the first step (1, 2, or 3 responses), “impaired responsiveness” is most likely from examination of 3>2>1 responses, on the second step: 6>5>4 responses, on the third step: 9>8>7 responses, on the fourth step: 12>11>10 responses, on the fifth step: 15>14>13 responses, on the sixth step: 18>17>16 responses, on the seventh step: 21>20>19 responses, and on the eighth steps: 23>22 responses (the used vaccine typically contains 23 different polysaccharides wherefore 23 responses is the upper limit). Thus, *within* any of these steps, an increase in number of examined responses causes an increased probability of fulfilling the criterion for impaired responsiveness.

The influence on “impaired responsiveness” probability is less straightforward when numbers of responses from different steps are compared. The key is in the probabilities for producing sufficient individual responses. **Figure S1** illustrates the relation in fictive populations with varying average probability of producing insufficient responses ($k$).

When $k$ is low (<0.35), then examination of more responses will tend to *decrease* the probability of “impaired responsiveness” (**A-C** in **Figure S1**). $k$ is decreased by testing persons with fairly intact ability to produce polysaccharide antibodies, by examining responses to the more immunogenic polysaccharides (for instance serotypes 14, 19F, and 19A, **Figure 3B** main text) and by applying methodologies providing higher IgG concentration estimates (for instance fluorescence bead-based immunoassays instead of ELISA [2]). When $k$ is high (>0.35), then examination of more responses will tend to *increase* the probability of “impaired responsiveness” (**E** in **Figure S1**). $k$ is increased by testing persons with fairly deficient ability to produce polysaccharide antibodies, by examining responses to the less immunogenic polysaccharides (for instance serotypes 4, 1, and 9V **Figure 3B** main text) and by applying methodologies providing lower IgG concentration estimates (for instance ELISA instead of fluorescence bead-based assays [2]).

For persons aged 2 years to 5 years, consensus guidelines define impaired responsiveness as sufficient responses to less than 50% of tested polysaccharides [1]. This definition entails a relationship much similar to the one discussed above; but the “steps” contain two consecutive numbers of responses instead of three.

References

1. F.A. Bonilla, I.L. Bernstein, D.A. Khan, Z.K. Ballas, J. Chinen, M.M. Frank, L.J. Kobrynski, A.I. Levinson, B. Mazer, R.P. Nelson Jr, J.S. Orange, J.M. Routes, W.T. Shearer, R.U. Sorensen, American Academy of Allergy, Asthma and Immunology, American College of Allergy, Asthma and Immunology, Joint Council of Allergy, Asthma and Immunology, Practice parameter for the diagnosis and management of primary immunodeficiency, Ann Allergy Asthma Immunol 94 (2005) S1-63.

2. M.J. Whaley, C. Rose, J. Martinez, G. Laher, D.L. Sammons, J.P. Smith, J.E. Snawder, R. Borrow, R.E. Biagini, B. Plikaytis, G.M. Carlone, S. Romero-Steiner, Interlaboratory comparison of three multiplexed bead-based immunoassays for measuring serum antibodies to pneumococcal polysaccharides, Clin Vaccine Immunol 17 (2010) 862-869.

**Figure S1. The probability of insufficient individual responses´ impact on impaired responsiveness frequency.**

Illustration of impaired responsiveness frequency in fictive populations with different *k*-values as functions of the numbers of responses examined. The presented *k*-values are products of the average *k*-value in the present study (0.23) multiplied by the following multiplicands: **A**=1/2, **B**=2/3, **C**=1, **D**=3/2, and **E**=2). Impaired responsiveness frequency was calculated as the percentage of the population expected to respond insufficiently to at least 30% of polysaccharides (under the defined conditions). Thus, the percentage expected to have impaired responsiveness when number of responses belonging to “first step” (i.e. 1, 2, or 3 responses) is calculated as 1 minus the probability of producing maximal number of insufficient responses without qualifying for impaired responsiveness. Thus, on the “first step”: $P(impaired\_responsiveness)=1-P(m=0)$ where $m$ is the number of maximal allowed insufficient responses without qualifying for impaired responsiveness (on the first step no insufficient responses are allowed). An additional subtrahend is added for every subsequent “step”; namely $P(m=1)$ on the “second step” and $P(m=2)$ on the “third step” and so forth. Each subtrahend is given by: $k^{m}\cdot\left(1-k\right)^{n-m}\cdot n!/m!/\left(n-m\right)!$ where $n$ is the number of responses.
